# Ankle-Brachial Index and Cardio-Ankle Vascular Index as Predictors of Cognitive Decline Over Time After Carotid Endarterectomy

**DOI:** 10.7759/cureus.26534

**Published:** 2022-07-03

**Authors:** Yuichiro Miyamatsu, Akira Nakamizo, Toshiyuki Amano, Satoshi Matsuo, Takahiro Kuwashiro, Masahiro Yasaka, Yasushi Okada, Masahiro Mizoguchi, Koji Yoshimoto

**Affiliations:** 1 Department of Neurosurgery, Clinical Research Institute, National Hospital Organization Kyushu Medical Center, Fukuoka, JPN; 2 Department of Neurosurgery, Graduate School of Medical Sciences, Kyushu University, Fukuoka, JPN; 3 Department of Cerebrovascular Medicine and Neurology, Clinical Research Institute, National Hospital Organization Kyushu Medical Center, Fukuoka, JPN

**Keywords:** frontal assessment battery, fab, executive function, cognitive function, cognistat, carotid endarterectomy, cavi, cardio-ankle vascular index, ankle-brachial index, abi

## Abstract

Objective: Patients with carotid stenosis risk cognitive impairment even after carotid endarterectomy (CEA) because of the long-term presence of vascular risk factors. Early prediction of cognitive decline is useful because early appropriate training for impaired cognitive domains can improve their functions. Ankle-brachial index (ABI) and cardio-ankle vascular index (CAVI) are frequently used as general indicators of systemic atherosclerosis and are associated with cognitive function in the general population. This study aimed to evaluate the utility of those vascular biomarkers for predicting cognitive decline in patients after CEA.

Methods: Patients who had undergone both CEA at our institute and cognitive evaluations between March 2016 and January 2022 were invited to participate in this study. Associations between ABI or CAVI three years before baseline and cognitive function at baseline were assessed retrospectively in 94 patients, and associations between ABI or CAVI at baseline and three-year changes in cognitive functions were assessed prospectively in 24 patients. Cognitive functions were assessed using the Frontal Assessment Battery (FAB) and Neurobehavioral Cognitive Status Examination (Cognistat).

Results: Low ABI three years before baseline was associated with poor performances on Cognistat and FAB at baseline. ABI, as a continuous measure, three years before baseline, showed positive linear associations with total Cognistat score and subscores for naming, construction, and judgment at baseline. The Wilcoxon signed-rank test showed that the total Cognistat score, total FAB score, and subscores for attention and inhibitory control declined after three years. CAVI at baseline was negatively associated with three-year changes in total Cognistat score and subscores for naming, construction, and memory.

Conclusion: Cognitive function can decline over time in patients with carotid stenosis even after CEA. ABI and CAVI might be useful to predict cognitive function and its decline among patients who have undergone CEA.

## Introduction

Carotid stenosis is a phenotype of atherosclerosis that is associated with vascular risk factors and relates to cognitive impairment [1-3]. Increased intima-media thickness (IMT), atherosclerotic plaques, and asymptomatic carotid stenosis are useful to detect individuals with cognitive impairment among the general population [4]. Cognitive function can be either improved or deteriorated in patients with severe carotid stenosis who have undergone carotid endarterectomy (CEA). This is because CEA can remove carotid plaque responsible for cerebral hypoperfusion and microembolism, but intraoperative microembolisms during the procedure, hypoperfusion during carotid clamping, and postoperative hyperperfusion syndrome can contribute to cognitive declines. Cognitive function can further decline over time even after CEA because those patients have been and will be further exposed to multiple vascular risk factors over a long period. Useful markers to predict cognitive declines after CEA may thus be useful to improve functional outcomes since cognitive functions might be maintained or improved if training appropriate for the impaired cognitive domain can be provided [5].

The ankle-brachial index (ABI) and cardio-ankle vascular index (CAVI) are widely accepted as vascular biomarkers for cardiovascular disease and are associated with cognitive function in the general population [6-14]. Most cross-sectional studies have shown that ABI is associated with cognitive function, while whether ABI can predict future cognitive decline remains controversial. Arterial stiffness, as represented by brachial-ankle pulse wave velocity (baPWV), is associated with cognitive function in both the elderly community-dwelling population and the primary care population [15-17]; however, baPWV is susceptible to blood pressure at measurement. Therefore, baPWV is not suitable for patients who are taking antihypertensive medication because arterial stiffness is underestimated in such patients [8]. On the other hand, CAVI represents arterial stiffness independently of blood pressure [18] and thus might be suitable for the patients who had undergone CEA in whom most patients take antihypertensive agents.

The aim of this study was to evaluate the usefulness of ABI and CAVI for predicting cognitive function in patients who had undergone CEA. We analyzed associations between ABI or CAVI at three years prior to baseline and cognitive function at baseline, and associations between ABI or CAVI at baseline and changes to cognitive functions at three years after baseline.

## Materials and methods

Patients

Between March 2016 and January 2022, a total of 129 patients who had undergone CEA at our institute underwent cognitive evaluations. Among these, ABI and CAVI were measured at three years prior to cognitive examination in 94 patients, and cognitive reassessments were performed three years later in 24 patients.

Cognitive examinations

The Japanese versions of the Neurobehavioral Cognitive Status Examination (Cognistat) [19] and Frontal Assessment Battery (FAB) [20] were adopted to assess neurocognitive and executive functions, respectively. Cognistat uses independent tests to evaluate the functioning of language, constructions, memory, calculations, and reasoning. The examination separately assesses the level of consciousness, orientation, and attention. Cognistat is typically utilized for assessing five major neurocognitive domains: language, visuospatial functioning, memory, arithmetic, and verbal reasoning [21]. This tool uses a screen-metric method and consists of 10 components: orientation, attention, comprehension, repetition, naming, construction, memory, calculation, similarities, and judgment [21]. A lower score indicates a greater degree of neurobehavioral cognitive impairment. The frontal lobes control conceptualization and abstract reasoning, mental flexibility, motor programming and executive control of action, resistance to interference, self-regulation, inhibitory control, and environmental autonomy. The FAB consists of six components: conceptualization, mental flexibility, programming, sensitivity to interference, inhibitory control, and environmental autonomy [22]. These subtests are chosen because the score of each of them significantly correlated with frontal metabolism, as measured in terms of the regional distribution of ^18^F-fluorodeoxyglucose in a PET study of patients with frontal lobe damage of various etiologies. Again, a lower score indicates a greater degree of executive dysfunction. A trained rater, blinded to the study aims, administered the neurocognitive examinations to participants. Among the 94 patients who underwent cognitive examinations at baseline and measurements of ABI and CAVI three years earlier, eight patients did not complete Cognistat, and one did not complete the FAB.

ABI and CAVI

Both ABI and CAVI were determined using a VaSera VS-1500 series (Fukuda Denshi, Tokyo, Japan). To calculate the ABI, brachial pressure and ankle pressure were measured on both the left and right limbs with the subject supine. Ankle pressures for each leg were then divided by the higher brachial systolic pressure. The lowest value for ABI [23,24] and the highest value for CAVI were taken. The formula for measuring CAVI is: CAVI = a{(2ρ/ΔP)×In(Ps/Pd)PWV(2)}+b [18]. The PWV from the heart to the ankle was calculated by measuring the length from the aortic valve to the ankle and dividing by the time interval between heart sounds and rises of the brachial and ankle pulse waves. Blood pressure was also measured at the brachial artery. Ps and Pd are the systolic and diastolic blood pressures, respectively, ΔP is Ps - Pd, ρ is blood density, and a and b are constants. 

Statistical analysis

Age is provided as mean ± standard deviation. Other data are provided as the median and interquartile range (IQR) unless otherwise indicated. Statistical analysis was performed using the Wilcoxon rank-sum test for non-parametric data. Differences in binary variables were assessed using Pearson’s chi-square test. The Wilcoxon signed-rank test was used to evaluate differences in test scores between baseline and three years later. Linear regression analysis was performed for paired ABI or CAVI three years prior and cognitive test scores at baseline, or ABI or CAVI at baseline and three-year changes in cognitive test scores. Differences were considered significant for values of p<0.05. All statistical analyses were performed using JMP version 16.0 software (SAS Institute, Cary, NC).

## Results

Patients

Table 1 summarizes the distribution of the cohort at baseline as stratified by ABI three years before baseline. Low ABI was more prevalent among patients with atherosclerosis obliterans, ischemic heart disease, lower Barthel index, and lower education.

**Table 1:** Baseline cohort characteristics stratified by ABI 3 years prior to baseline ICA: internal carotid artery; WMH: white matter hyperintensity; PVH: periventricular hyperintensity. * p<0.05; † p<0.01; ‡ p<0.001; § p<0.0001

	ABI ≥ 0.9 (n=69)	ABI < 0.9 (n=25)	p
Age (years)	76.9 ± 6.1	77.1 ± 5.4	0.7414
Male (%)	59 (85.5)	19 (76.0)	0.2785
Symptomatic presentation (%)	44 (63.8)	15 (60.0)	0.7385
Medical history (%)			
Hypertension	64 (92.8)	23 (92.0)	0.9021
Diabetes mellitus	29 (42.3)	13 (52.0)	0.3903
Dyslipidemia	62 (89.9)	24 (96.0)	0.3455
Chronic kidney disease	13 (18.8)	8 (32.0)	0.1759
Atherosclerosis obliterans	1 (1.5)	9 (36.0)	<0.0001§
Ischemic heart disease	22 (31.9)	17 (68.0)	0.0017†
Atrial fibrillation	4 (5.8)	1 (4.0)	0.7316
Brain infarction	12 (17.4)	7 (28.0)	0.2578
Smoking history	51 (73.9)	18 (72.0)	0.8529
Current smoking	6 (8.7)	5 (20.0)	0.1319
Alcohol > 40 g/day	2 (2.9)	3 (12.0)	0.0823
Deep WMH Fazekas grade	1 (0, 1.75)	1 (1, 2. 5)	0.1657
PVH Fazekas grade	1 (0, 2)	1 (0, 2)	0.8596
Education (years)	12 (12, 16)	12 (9, 13.5)	0.0182*
Mini-Mental State Examination	28 (25, 29)	27 (24, 29.5)	0.8343
Modified Rankin scale	0 (0, 1)	0 (0, 2)	0.1102
Barthel index	100 (100, 100)	100 (97.5, 100)	0.0053†
Operated side (%)			0.1269
Right	38 (55.1)	14 (56.0)	
Left	28 (40.6)	7 (28.0)	
Bilateral	3 (4.4)	4 (16.0)	

Associations between ABI or CAVI three years before baseline and cognitive functions at baseline

**Figure 1:**
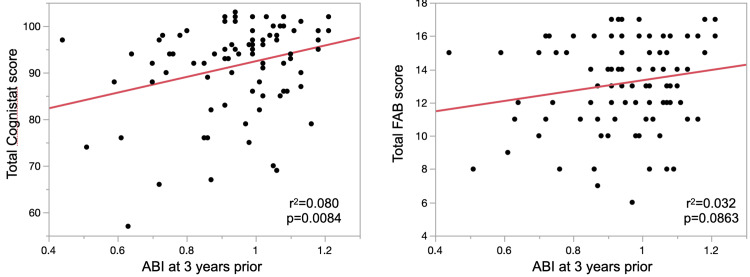
Linear regression of ABI at three years prior to baseline and total Cognistat score (left) or total FAB score (right) at baseline. ABI: ankle-brachial index; FAB: frontal assessment battery

Table 2 summarizes test scores at baseline stratified by ABI three years before baseline. Lower ABI at three years before baseline was associated with poor performance on total Cognistat score, repetition, construction, similarity, judgment, conceptualization, mental flexibility, and sensitivity to interference. Figure 1 shows the linear relationship between ABI, as a continuous measure, three years before baseline and the total Cognistat score at baseline. ABI at three years before baseline was also associated with the Cognistat subscores for naming, construction, and judgment at baseline (r^2^=0.048, p=0.0434; r^2^=0.093, p=0.0044; and r^2^=0.088, p=0.0057; respectively). CAVI three years prior was not associated with any Cognistat or FAB scores or subscores.

**Table 2:** Cognitive test scores stratified by ABI three years prior to baseline * p<0.05; † p<0.01

	ABI ≥ 0.9	ABI < 0.9	p
Cognistat			
Total score	96 (90, 99.25)	91 (76, 94)	0.0022†
Orientation	10 (10, 10)	10 (9, 10)	0.1173
Attention	10 (7.5, 10)	10 (6, 10)	0.3387
Comprehension	10 (10, 10)	10 (7, 10)	0.1297
Repetition	11 (9, 11)	9.5 (7, 11)	0.0467*
Naming	10 (10, 10)	10 (9, 10)	0.0868
Construction	9 (8, 11)	8 (7.25, 9)	0.0189*
Memory	8 (6, 9.25)	8 (6.25, 9)	0.4128
Calculation	10 (10, 10)	10 (8, 10)	0.0829
Similarity	10 (9, 10)	9 (9, 10)	0.0022†
Judgment	10 (10, 11)	9 (9, 10)	0.0012†
Frontal assessment battery			
Total score	14 (12, 16)	12 (10, 15)	0.0544
Conceptualization	2 (2, 2)	2 (1, 2)	0.0063†
Mental flexibility	2 (2, 3)	2 (1, 2)	0.0321*
Programming	2 (1, 3)	2 (1, 3)	0.9963
Sensitivity to interference	3 (3, 3)	3 (1.5, 3)	0.0121*
Inhibitory control	2 (1, 3)	3 (1, 3)	0.3193
Environmental autonomy	3 (3, 3)	3 (3, 3)	0.0991

Associations between ABI or CAVI at baseline and three-year changes in cognitive functions

**Figure 2:**
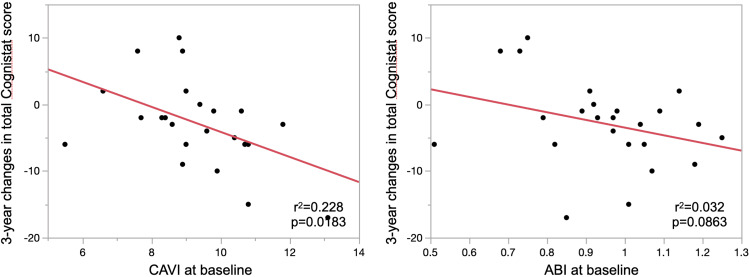
Linear regression of CAVI (left) or ABI (right) at baseline and three-year changes in total Cognistat score. CAVI: cardio-ankle vascular index; ABI: ankle-brachial index

Table 3 demonstrates three-year changes in cognitive test scores. The Wilcoxon signed-rank test showed that the total Cognistat score, total FAB score, and subscores for attention and inhibitory control declined after three years. Two patients experienced ≥5 points decline in the total FAB score, and three patients experienced ≥10 points decline in total Cognistat score three years later. The decline in total FAB score ≥5 points was associated with a decline in subscores for conceptualization and sensitivity to interference (p=0.0188 and 0.0286, respectively). The decline in total Cognistat score ≥10 points was associated with a decline in subscores for orientation, comprehension, and naming (p=0.0296, 0.0098, and 0.0158, respectively). Figure 2 shows a negative linear association between CAVI at baseline and three-year changes in total Cognistat score. CAVI at baseline was also negatively associated with three-year changes in Cognistat subscores for naming, construction, and memory (r^2^=0.176, p=0.0414; r^2^=0.194, p=0.0314; and r^2^=0.364, p=0.0018; respectively). CAVI at baseline was not associated with three-year changes in the total FAB score (r2=0.0002, p=0.9454). ABI at baseline was not associated with three-year changes in any Cognistat or FAB scores or subscores.

**Table 3:** Cognitive test scores at baseline and three years later * p<0.05

	Baseline	3 years later	p	Change
Cognistat				
Total score	96 (90.25, 100)	92 (86.25, 98.25)	0.0240*	-2.5 (-6, -0.25)
Orientation	10 (10, 10)	10 (9.25, 10)	0.3000	0 (0, 0)
Attention	10 (8, 10)	9 (6, 10)	0.0435*	0 (-2.75, 0)
Comprehension	10 (10, 10)	10 (10, 10)	0.6361	0 (0, 0)
Repetition	11 (9, 11)	10 (8, 11)	0.0514	0 (-1, 0)
Naming	10 (10, 10)	10 (9.25, 10)	0.9561	0 (0, 0)
Construction	9 (8, 11)	8.5 (7, 11)	0.1915	0 (-2, 0.75)
Memory	9 (7, 9.75)	8.5 (6.25, 9)	0.3626	0 (-1, 1)
Calculation	10 (10, 10)	10 (10, 10)	0.5583	0 (0, 0)
Similarity	9.5 (9, 10)	9.5 (9, 10)	0.9415	0 (0, 0)
Judgment	10 (9, 11)	10 (10, 11)	0.9318	0 (-1, 1)
Frontal assessment battery				
Total score	14 (12, 15)	13 (11, 14)	0.0145*	-1 (-2, 0)
Conceptualization	2 (2, 2)	2 (1, 2)	0.3896	0 (-1, 0)
Mental flexibility	2 (2, 2)	2 (2, 3)	0.6668	0 (-1, 1)
Programming	2 (1, 3)	2 (1, 3)	0.6745	0 (-1, 0)
Sensitivity to interference	3 (3, 3)	3 (2, 3)	0.2023	0 (-1, 0)
Inhibitory control	2 (1, 3)	1 (1, 2)	0.0310*	-1 (-2, 0)
Environmental autonomy	3 (3, 3)	3 (3, 3)	1.0	0 (0, 0)

## Discussion

We showed that low ABI three years prior was associated with poor cognitive performance at baseline, and CAVI was negatively associated with three-year changes in cognitive function among patients who had undergone CEA. This suggests that ABI and CAVI help predict individuals with cognitive decline among cohorts with a higher prevalence of vascular risk factors than the general population. Our results demonstrated that specific cognitive domains, including executive functions, were impaired among patients with lower ABI three years prior. This is consistent with previous reports showing that patterns of cognitive impairment among patients with low ABI resembled those of vascular dementia, in which executive function was more impaired and verbal memory was less impaired [11-13]. The association between lower ABI and cognitive impairment is strongest among the oldest individuals, a finding that has been attributed to longer exposures to vascular risk factors [10]. Vascular risk factors, including past and current smoking, male sex, coronary heart disease, cerebrovascular disease, physical inactivity, and hypercholesterolemia, were significantly associated with the prevalence of carotid plaque in a population-based Norwegian study of 3683 participants between 63 and 65 years old [1]. Midlife vascular risk factors, including smoking, diabetes, hypertension, and prehypertension, were associated with the incidence of dementia 25 years later in the Atherosclerosis Risk in Communities cohort [2]. The Baltimore Longitudinal Study of Aging showed that carotid IMT at baseline was associated with declines in memory, fluency, and executive function at a mean of four years later in participants without any history of cardiovascular disease, cerebrovascular disease, diabetes, or carotid endarterectomy [3].

Several studies have assessed associations between di- or trichotomous ABI and cognitive functions in the general population. The Lifestyle Interventions and Independence for Elders (LIFE) trial demonstrated an association between lower ABI and poorer performance on the modified Mini-Mental State Examination (3MSE, global cognitive function), Digit Symbol (processing speed), Hopkins Verbal Learning Test (episodic memory), flanker task (selective attention and response inhibition), and task switching (attentional flexibility) in a cohort of 1601 sedentary and functionally limited subjects between 70 and 89 years old [25]. The US National Health and Nutrition Examination Study demonstrated that scores on Digit Symbol (processing speed) were higher among subjects with ABI <0.90 than among those with ABI ≥0.90 in 2386 participants > 60 years old [26]. In contrast, a population-based study revealed no significant group differences in cognitive functioning (nonverbal reasoning, verbal fluency, working memory, processing speed) between ABI <0.90 and ≥0.90, but a continuous measure of ABI revealed associations with general cognition and processing speed [10]. Associations between ABI at baseline and future cognitive function have also been assessed in the general population. The Edinburgh Artery Study demonstrated that ABI ≤0.95 at baseline was associated with lower scores on Raven’s Matrices (odds ratio (OR) 1.6), verbal fluency (OR 1.8), and Digit Symbol (OR 2.3) after 10 years [11]. The Cardiovascular Health Study in the United States revealed that individuals with ABI <0.90 at baseline experienced a mean 4.62-point decline on 3MSE and a mean 2.73-point decline in Digit Symbol over the seven years, whereas those with ABI ≥0.90 experienced only mean declines of 0.66 and 0.39, respectively [14]. Conversely, the LIFE study demonstrated that baseline ABI, as a continuous measure, was not associated with overall changes in cognitive function, and changes in ABI were not associated with changes in cognitive function over two years [25]. The Edinburgh Artery Study at the 15-year follow-up also showed that continuous measures of ABI were not associated with changes in cognitive function [27]. Whether ABI can predict future cognitive decline thus remains controversial. In contrast to the general population, the utility of ABI in predicting cognitive decline among patients possessing various vascular risk factors has not been elucidated because the association between ABI and cognitive function is weakened in participants with multiple other risk factors [25,28]. Our results indicated that ABI <0.9 at three years before baseline was associated with poorer cognitive functions at baseline and that continuous measures of ABI three years before baseline displayed a linear association with total cognition at baseline in patients who had undergone CEA.

CAVI indicates blood pressure-independent arterial stiffness, unlike baPWV, and correlates better with parameters of left ventricular diastolic indices, low-density lipoprotein cholesterol, and angina pectoris than the baPWV [29]. CAVI remained associated with MMSE even after adjusting for age, height, weight, and sex in a cross-sectional study of community-dwelling subjects [8]. A possible mechanism underlying the association between arterial stiffness and cognitive decline is that the stiffening of an artery reduces its buffering capacity to resist increased pulse waves, resulting in damage to small cerebral vessels [30]. In the present study, CAVI was negatively associated with three-year changes in total cognition, naming, construction, and memory in patients who had undergone CEA, suggesting that CAVI may prove helpful for predicting future cognitive declines even in patients with multiple vascular risk factors.

Some limitations need to be considered when interpreting the results. First, the sample size was relatively small. Second, the observational period was relatively short. Third, this study did not include a control group. The present findings might depict generalizable observations.

## Conclusions

In this study, associations between ABI or CAVI three years before baseline and cognitive function at baseline were assessed retrospectively in 94 patients, and associations between ABI or CAVI at baseline and three-year changes in cognitive functions were assessed prospectively in 24 patients. Low ABI three years before baseline was associated with poor cognitive performance at baseline, and CAVI at baseline was negatively associated with three-year changes in cognitive function among patients who had undergone CEA. ABI and CAVI may offer a useful marker to predict cognitive function and its future declines among patients with a history of CEA who have been and will be exposed to various vascular risk factors related to cognitive declines. Early detection of declines in specific cognitive domains might be important since cognitive functions might be maintained or improved if training appropriate for the impaired cognitive domains can be provided.
